# [^18^F]DCFPyL PET/CT versus [^18^F]fluoromethylcholine PET/CT in Biochemical Recurrence of Prostate Cancer (PYTHON): a prospective, open label, cross-over, comparative study

**DOI:** 10.1007/s00259-023-06301-5

**Published:** 2023-06-21

**Authors:** Daniela-Elena Oprea-Lager, Eric Gontier, Lina García-Cañamaque, Mathieu Gauthé, Pierre Olivier, Mercedes Mitjavila, Pilar Tamayo, Philippe Robin, Ana Maria García Vicente, Anne-Charlotte Bouyeure, Alban Bailliez, Antonio Rodríguez-Fernández, Sinan Ben Mahmoud, Juan Antonio Vallejo-Casas, Philippe Maksud, Charles Merlin, Paul Blanc-Durand, Clément Drouet, Hubert Tissot, Irina Vierasu, Thierry Vander Borght, Evelyne Boos, Florence Chossat, Marina Hodolic, Caroline Rousseau

**Affiliations:** 1 Department of Radiology & Nuclear Medicine, Amsterdam University Medical Centers, VU University Medical Center, Cancer Center Amsterdam, De Boelelaan 1117, 1081 HV Amsterdam, The Netherlands; 2 Service de Médecine Nucléaire, Centre de Cancérologie de La Sarthe, Le Mans, France; 3 Servicio de Medicina Nuclear, Grupo HM Hospitales, Universidad CEU San Pablo, Madrid, Spain; 4 Service de Médecine Nucléaire, Hôpital Tenon, Paris, France; 5 Service de Médecine Nucléaire, CHRU, Nancy, France; 6 Servicio de Medicina Nuclear, Hospital Universitario Puerta de Hierro Majadahonda, Madrid, Spain; 7 Servicio de Medicina Nuclear, IBSAL, Hospital Universitario de Salamanca, Salamanca, Spain; 8 Service de Médecine Nucléaire, Centre Hospitalier Universitaire de Brest, Brest, France; 9 UMR 1304, Inserm, Univ Brest, CHRU Brest, GETBO, Brest, France; 10 Servicio de Medicina Nuclear, Hospital General Universitario de Ciudad Real, Ciudad Real, Spain; 11 Service de Médecine Nucléaire, Centre Henri Becquerel, Rouen, France; 12 Service de Médecine Nucléaire Humanitep, Groupement Des Hôpitaux de L’Institut Catholique de Lille, Hôpital Saint-Philibert, Lomme, France; 13 Service de Médecine Nucléaire, Hôpital Privé Le Bois, Iris Imagerie, Lille, France; 14 Servicio de Medicina Nuclear, Hospital Universitario Virgen de Las Nieves, Granada, Spain; 15 Instituto de Investigación Biosanitaria IBS, Granada, Spain; 16 Service de médecine nucléaire, Hôpital de Mercy, CHR Metz-Thionville, Thionville, France; 17 UGC Medicina Nuclear, Instituto Maimónides de Investigación Biomédica de Córdoba (IMIBIC), Hospital Universitario Reina Sofía, Córdoba, Spain; 18 Service de médecine nucléaire Hôpital de la Pitié-Salpétriére, Sorbonne-Université, Paris, France; 19 Service de médecine nucléaire, Centre Jean Perrin, Clermont-Ferrand, France; 20 Imagerie moléculaire et stratégies théranostiques, UMR1240, Université Clermont Auvergne, Inserm, Clermont-Ferrand, France; 21 Service de médecine nucléaire, CHU H. Mondor, Créteil, France; Université Paris Est Créteil (U-PEC), Créteil, France; 22 Service de médecine nucléaire, Centre Georges-François-Leclerc, Dijon, France; 23 Service de médecine nucléaire, Institut Curie, Paris, France; 24 Department of Nuclear Medicine, HUB, Hôpital Erasme Université libre de Bruxelles (ULB), Brussels, Belgium; 25 Service de médecine nucléaire, CHU UCL Namur, UCLouvain, Godinne, Belgium; 26 Curium, Paris, France; 27 Univ Nantes, Univ Angers, INSERM, CNRS, CRCI2NA, Nantes, France; 28 Service de médecine nucléaire, Institut de cancérologie de l’Ouest, Saint-Herblain, France

**Keywords:** [^18^F]DCFPyL, Piflufolastat, PYLCLARI, PSMA PET/CT, Biochemical recurrence, Prostate cancer

## Abstract

**Purpose:**

Primary objective was to compare the per-patient detection rates (DR) of [^18^F]DCFPyL versus [^18^F]fluoromethylcholine positron emission tomography/computed tomography (PET/CT), in patients with first prostate cancer (PCa) biochemical recurrence (BCR). Secondary endpoints included safety and impact on patient management (PM).

**Methods:**

This was a prospective, open label, cross-over, comparative study with randomized treatment administration of [^18^F]DCFPyL (investigational medicinal product) or [^18^F]fluoromethylcholine (comparator). Men with rising prostate-specific antigen (PSA) after initial curative therapy were enrolled. [^18^F]DCFPyL and [^18^F]fluoromethylcholine PET/CTs were performed within a maximum time interval of 12 days. DR was defined as the percentage of positive PET/CT scans identified by 3 central imaging readers. PM was assessed by comparing the proposed pre-PET/CT treatment with the local treatment", defined after considering both PET/CTs.

**Results:**

A total of 205 patients with first BCR after radical prostatectomy (73%; median PSA = 0.46 ng/ml [CI 0.16;27.0]) or radiation therapy (27%; median PSA = 4.23 ng/ml [CI 1.4;98.6]) underwent [^18^F]DCFPyL- and/or [^18^F]fluoromethylcholine -PET/CTs, between July and December 2020, at 22 European sites. 201 patients completed the study. The per-patient DR was significantly higher for [^18^F]DCFPyL- compared to [^18^F]fluoromethylcholine -PET/CTs (58% (117/201 patients) vs. 40% (81/201 patients), *p* < 0.0001). DR increased with higher PSA values for both tracers (PSA ≤ 0.5 ng/ml: 26/74 (35%) vs. 22/74 (30%); PSA 0.5 to ≤ 1.0 ng/ml: 17/31 (55%) vs. 10/31 (32%); PSA 1.01 to < 2.0 ng/ml: 13/19 (68%) vs. 6/19 (32%);PSA > 2.0: 50/57 (88%) vs. 39/57 (68%) for [^18^F]DCFPyL- and [^18^F]fluoromethylcholine -PET/CT, respectively). [^18^F]DCFPyL PET/CT had an impact on PM in 44% (90/204) of patients versus 29% (58/202) for [^18^F]fluoromethylcholine. Overall, no drug-related nor serious adverse events were observed.

**Conclusions:**

The primary endpoint of this study was achieved, confirming a significantly higher detection rate for [^18^F]DCFPyL compared to [^18^F]fluoromethylcholine, in men with first BCR of PCa, across a wide PSA range. [^18^F]DCFPyL was safe and well tolerated.

**Supplementary information:**

The online version contains supplementary material available at 10.1007/s00259-023-06301-5.

## Introduction

Prostate cancer (PCa) is the most common type of malignancy in men, accounting for 10% of all cancer deaths among men within the European Union (EU) [1]. Most patients with metastatic PCa succumb to disease [2]. A rising of the prostate-specific antigen (PSA) tumour marker, after initial therapy, suggests biochemical recurrence (BCR). This may occur in 20–30% of PCa patients within five years, before metastatic disease can be established by conventional imaging modalities [3–7].

Correct localisation of BCR is essential for further treatment planning, since potentially curative treatment is feasible in local recurrence or locoregional lymph node metastases, whereas in distant metastases, (palliative) systemic treatment should be considered. Moreover, there is currently an increasing interest in metastasis-directed therapies (MDTs) in patients with minimal metastatic tumour burden (oligometastatic disease) [8–10].

[^18^F]fluoromethylcholine has been approved in the EU since 2010 for the detection of recurrent PCa in patients with rising PSA. However, [^18^F]fluoromethylcholine has clinical impact mainly when PSA exceeds 4 ng/mL [11]. The introduction of a new class of positron emission tomography (PET) radiopharmaceuticals targeting the prostate-specific membrane antigen (PSMA), has facilitated the detection of recurrent or metastatic PCa cells that is otherwise occult on conventional imaging [12–15]. PSMA is a transmembrane glycoprotein expressed by the majority of PCa, with proven high detection rate at early BCR, which led PSMA PET to a grade A recommendation by the European Guidelines of PCa [16].

Various radiolabelled anti-PSMA small molecules and monoclonal antibodies have been used to detect PCa recurrence. ^18^F-Piflufolastat, [^18^F]DCFPyL, is a radiolabelled small-molecule that binds to the extracellular domain of PSMA with high affinity and is approved for staging of primary and recurrent disease by the US FDA. Compared to ^68^ Ga, ^18^F has the advantage of a shorter positron range, longer half-life (110 vs. 68 min), possibility of large-scale production, and ease of regional distribution. Over the last years, different radiopharmaceuticals targeting PSMA have been synthesised and used in academic investigator-sponsored studies and on compassionate use [17–19]. However, the performance of [^18^F]DCFPyL, compared to [^18^F]fluoromethylcholine has not been formally studied.

In this study, we aimed to compare the per-patient detection rates (DR) of [^18^F]DCFPyL versus [^18^F]fluoromethylcholine positron emission tomography/ computed tomography (PET/CT), in patients with first BCR in PCa. Secondary endpoints included safety of [^18^F]DCFPyL and impact on patient management (PM).

## Methods

### Study design

This was a prospective, open label, cross-over, comparative study with randomised treatment administration of [^18^F]DCFPyL, as investigational medicinal product (IMP) and [^18^F]fluoromethylcholine, as comparator. [^18^F]DCFPyL- and [^18^F]fluoromethylcholine-PET/CT scans were performed on the same scanner, within a maximum time interval of 12 days.

External, independent coded, blinded and centralized interpretation of [^18^F]DCFPyL- and [^18^F]fluoromethylcholine-PET/CTs was performed by three independent, experienced nuclear medicine physicians who were not otherwise involved in the trial. The order of presentation of the PET/CTs with IMP and comparator was randomized, with a washout period of 2 weeks between the interpretation.

As part of the routine care practice, enrolled patients received appropriate treatment. Treatment decisions were pragmatic, at the discretion of the referring physician, based on all available clinical information, including local reports of both PET/CT scans and any other imaging findings. For the purpose of the study, patients were followed-up, up to 10 months after the second tracer injection, to provide any results of subsequent biopsies, imaging studies, clinical findings, PSA measurements, and disease management, if performed routinely.

Consensus on the disease status was obtained from a multidisciplinary independent board (truth panel), based on the surrogate standard of reference which included all available results. Assessments were made on a per-region and per-patient basis. Additionally, the truth panel assessed the impact of each PET/CT examination on disease restaging and change in treatment intent, by filling in a PM questionnaire.

### Patient population

Patients were enrolled between the 1^st^ of July 2020 and the 4^th^ of December 2020, from twenty-two sites in 4 European countries (France, Belgium, Spain and The Netherlands). Ethical approval was gained from all local institutional review boards. All patients signed written informed consent prior to participation in this study.

Inclusion criteria were: male, age ≥ 18 years, histopathological proven prostate adenocarcinoma, first suspected recurrence of PCa based on rising PSA after initial curative therapy like radical prostatectomy (RP), with PSA ≥ 0.2 ng/mL confirmed by a subsequent PSA determination, or radiation therapy (RT) with PSA ≥ 2 ng/mL above the nadir after therapy, regardless of the serum concentration of the PSA nadir.

Exclusion criteria were: ECOG status > 2; history of previous salvage therapies (including salvage radiotherapy or salvage lymph node dissection), adjuvant radiotherapy, cryotherapy or high-intensity focused ultrasound (HIFU); other active malignant tumour; treatment with: androgen deprivation therapy (ADT) in the past 30 days/ ongoing; colchicine in the past 8 days/ ongoing or hematopoietic colony stimulating factors (CSF) in the past 5 days/ ongoing.

### Sample size calculation

A sample size of 141 patients was calculated to achieve 90% power for showing a difference in per-patient detection rate of 12% between [^18^F]fluoromethylcholine- and [^18^F]DCFPyL-PET/CT at the two-sided type 1 error rate of 5%. The required sample size was set to 217 patients, assuming a rate of non-assessable patients among the randomised patients of approximately 35%.

### Imaging procedures

For both tracers, whole body PET-acquisition started from the mid-thigh to at least the base of the skull. PET scans were acquired in three-dimensional (3D) mode with an acquisition time of 3 min per bed position or equivalent for continuous acquisition. Overall, PET coverage was identical to the anatomical CT scan range. Low-dose CT acquisition of parameters (such as kV, mAs, pitch in helical CT, dose modulation, etc.) was performed in accordance with institutional protocols.

Acquisition procedure for [^18^F]fluoromethylcholine consisted of a dynamic PET acquisition over the pelvis including the prostate bed from 1 min post injection (p.i.) of 2 to 4 MBq/kg body weight (140–280 MBq) up to 9 min p.i. (8 images, 1 min each). Static whole-body PET acquisition followed, 20 min p.i. If there was any doubt concerning potential lesions with a slight uptake, a second static acquisition was allowed 60 min p.i.. For [^18^F]DCFPyL, a whole-body PET/CT was performed 120 (± 15) minutes p.i. of a standard dose of 330 MBq (300–360 MBq).

### Safety analysis

The safety analysis set included all randomised patients who received at least one injection of either study product (IMP or comparator), regardless of any protocol deviations. All potential adverse events (AEs) described for the 2 radiopharmaceuticals were collected from the time of the injection up to 24 h and they were all documented. For a detailed description, see Supplemental file 1.

### Statistical analyses

Analyses were performed mainly as descriptive statistics and were summarised depending on the nature of the analysed variables (see Supplemental file 2).

The analysis of the primary objective was performed in the full analysis set (FAS). The Prescott’s test was used to assess the difference between the two tracers in terms of detection. The per-patient detection rate was computed for each reader independently and the intra-reader variability and inter-reader variability were evaluated by the Cohen’s kappa coefficient and the Fleiss’ kappa coefficient, respectively, on the FAS (without any imputation). *P*-values ≤ 0.05 were considered statistically significant and were calculated only for the primary endpoint, without subgroup analysis, in order to avoid the tests multiplicity.

## Results

### Patients

**Fig. 1:**
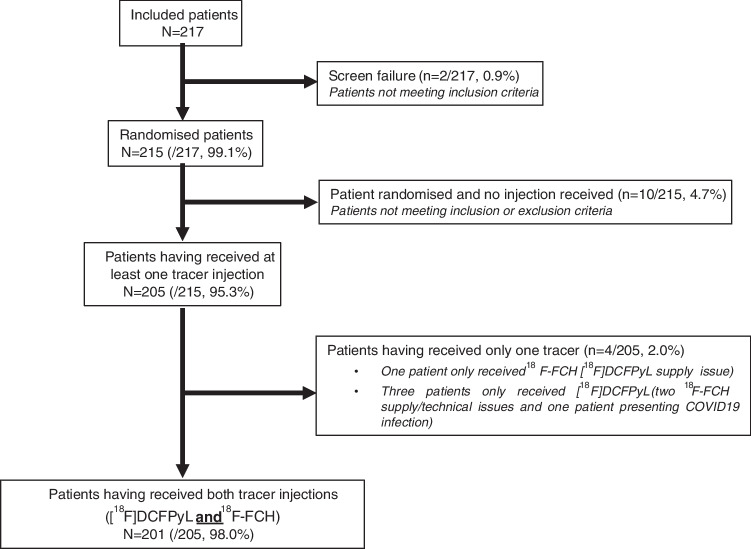
Patient flow diagram

Overall, 217 patients were enrolled. 201 out 217 patients (92.6%) met the definition for study completion and received both tracers. The reasons for 16 patients not completing the study were: PSA not measured on first PET/CT injection (n = 10), lost to follow-up (n = 1), disease progression (n = 1), COVID-19 disease (n = 1), and imaging/ technical issues (n = 3). Patient flow diagram is presented in Fig. 1.

Among 217 enrolled patients, 215/217 (99%) patients were randomised. Two-hundred-five out of 215 (95%) patients received at least one tracer injection (IMP or comparator) corresponding to 204 patients who received [^18^F]DCFPyL and 202 patients who received [^18^F]fluoromethylcholine. Two-hundred-one out of 205 (98.0%) patients received both tracers’ injections (IMP and comparator). Patient characteristics are provided in Table 1. Patients were followed-up for a period of up to 10 months after the second tracer injection.

**Table 1:** Patient characteristics

Characteristics	[^18^F]Choline PET/CT followed by [^18^F]DCFPyl PET/CT N = 102	[^18^F]DCFPyL PET/CT followed by [^18^F]Choline PET/CT N = 103	Total N = 205
Age at baseline visit (years)			
Mean ± SD	69.7 ± 6.4	70.3 ± 7.8	70.0 ± 7.1
Median [range]	70.0 [53-88]	71.0 [54-88]	71.0 [53-88]
BMI (kg/m^2^)			
Missing values	8	9	17
Mean ± SD	28.11 ± 4.23	27.20 ± 3.90	27.66 ± 4.08
Median [range]	27.85 [20.6–42.0]	26.84 [18.9–39.0]	27.39 [18.9–42.0]
ECOG performance status (%)			
Grade 0	98 (96.1)	91 (88.3)	189 (92.2)
Grade 1	4 (3.9)	10 (9.7)	14 (6.8)
Grade 2	0 (0.0)	2 (1.9)	2 (1.0)
Grade ≥ 3	0 (0.0)	0 (0.0)	0 (0.0)
Time since PCa diagnosis (months)			
Mean ± SD	60.21 ± 46.69	64.22 ± 52.60	62.23 ± 49.66
Median [range]	45.57 [2.5–207.7]	45.57 [5.1–214.1]	45.57 [2.5–214.1]
ISUP grade (%)			
Missing values	1	0	1
Grade 1	22 (21.8)	24 (23.3)	46 (22.5)
Grade 2	30 (29.7)	44 (42.7)	74 (36.3)
Grade 3	28 (27.7)	18 (17.5)	46 (22.5)
Grade 4	13 (12.9)	10 (9.7)	23 (11.3)
Grade 5	8 (7.9)	7 (6.8)	15 (7.4)
D’Amico risk classes (%)			
Missing values	1	0	1
Low risk	13 (12.9)	12 (11.7)	25 (12.3)
Intermediate risk	19 (18.8)	25 (24.3)	44 (21.6)
High risk	39 (38.6)	40 (38.8)	79 (38.7)
Not applicable	30 (29.7)	26 (25.2)	56 (27.5)
TNM stage			
Primary tumor (%)			
Missing values	3	1	4
T1a	0 (0.0)	1 (1.0)	1 (0.5)
T1b	0 (0.0)	1 (1.0)	1 (0.5)
T1c	14 (100.0)	6 (75.0)	20 (90.9)
T2a	14 (26.4)	23 (34.3)	37 (30.8)
T2b	5 (9.4)	9 (13.4)	14 (11.7)
T2c	34 (64.2)	35 (52.2)	69 (57.5)
T3a	11 (39.3)	11 (44.0)	22 (41.5)
T3b	17 (60.7)	14 (56.0)	31 (58.5)
Regional lymph nodes (%)			
Missing values	3	1	4
N0	64 (64.6)	60 (58.8)	124 (61.7)
N1	6 (6.1)	5 (4.9)	11 (5.5)
Nx	29 (29.3)	37 (36.3)	66 (32.8)
Initial treatment with curative intent (%)			
Radical prostatectomy +/- eLND	74 (72.5)	76 (73.8)	150 (73.2)
Radiation therapy	28 (27.5)	27 (26.2)	55 (26.8)
For patients treated with radical prostatectomy +/- eLND patients			
PSA at first PET/CT injection (ng/mL)			
Missing values	9	6	15
Mean ± SD	1.20 ± 3.38	0.67 ± 0.63	0.93 ± 2.39
Median [range]	0.47 [0.16–27.00]	0.46 [0.18–3.29]	0.46 [0.16–27.00]
PSA classes (ng/mL) (%)			
< 0.2	2 (3.1)	4 (5.7)	6 (4.4)
[0.2—0.5]	35 (53.8)	36 (51.4)	71 (52.6)
[0.51 – 1]	16 (24.6)	15 (21.4)	31 (23.0)
[1.01 – 2]	7 (10.8)	11 (15.7)	18 (13.3)
> 2	5 (7.7)	4 (5.7)	9 (6.7)
For patients treated with radiation therapy			
PSA at first PET/CT injection (ng/mL)			
Missing values	3	2	5
Mean ± SD	9.39 ± 19.31	6.07 ± 6.30	7.73 ± 14.32
Median [range]	4.07 [2.2–98.6]	4.38 [1.4–33.8]	4.23 [1.4–98.6]
PSA classes (ng/mL) (%)			
[1.01 – 2]	0 (0.0)	1 (4.0)	
> 2	25 (100.0)	24 (96.0)	1 (2.0) 49 (98.0)
PSA Doubling time (months) (%)			
Missing values	87	90	177
Mean ± SD	9.53 ± 10.17	12.02 ± 14.76	10.80 ± 12.74
Median [range]	6.88 [0.3–64.4]	6.92 [0.8–91.5]	6.90 [0.3–91.5]
≤ 6	39 (44.8)	36 (40.0)	75 (42.4)
> 6	48 (55.2)	54 (60.0)	102 (57.6)
Velocity (ng/mL/year)			
Missing values	15	13	28
Mean ± SD	5.45 ± 16.94	5.18 ± 25.58	5.31 ± 21.71
Median [range]	0.77 [0.04–120.40]	0.80 [0.02–239.18]	0.80 [0.02–239.18]
[18F]DCFPyL injected activity (MBq)			
Missing values	8	8	16
Mean ± SD	313.19 ± 31.92	320.00 ± 23.98	316.56 ± 28.40
Median [range]	318.03 [186.9–373.0]	324.94 [228.6–364.3]	321.19 [186.9–373.0]
Time between injection and DCFPyL PET/CT (min)			
Missing values	122.6 ± 7.5	120.6 ± 8.3	121.6 ± 7.9
Mean ± SD	120.0 [109–147]	120.0 [80–150]	120.0 [80–150]
Median [range]			

PSA: Prostate specific antigen; PCa: prostate cancer

### Primary endpoint

A total of 205 patients with first BCR underwent [^18^F]DCFPyL- and/or [^18^F]fluoromethylcholine-PET/CT. Among them, 150 patients (73%) were treated with curative intent by RP ± extended lymph node dissection (eLND) (median PSA at first injection = 0.46 ng/ml [0.2;27.0]), and 55 patients (27%) were treated by external-beam RT or brachytherapy (median PSA at first injection = 4.23 ng/ml [1.4;98.6]). The per-patient DR, based on the number of observed cases receiving at least one tracer (N = 205), is presented in Table 1.

The per-patient DR, based on the number of observed cases receiving both tracers (N = 201), was significantly higher for [^18^F]DCFPyL compared to [^18^F]fluoromethylcholine-PET/CT (58% (117/201 patients) vs. 40% (81/201 patients), *p* < 0.0001). DR increased numerically with higher PSA values for both tracers (PSA ≤ 0.5 ng/ml: 26/74 (35%) vs. 22/74 (30%); PSA 0.5 to ≤ 1.0 ng/ml: 17/31 (55%) vs. 10/31 (32%); PSA 1.01 to < 2.0 ng/ml: 13/19 (68%) vs. 6/19 (32%); PSA > 2.0: 50/57 (88%) vs. 39/57 (68%) for [^18^F]DCFPyL- and [^18^F]fluoromethylcholine-PET/CT, respectively). DR according to the PSA concentration level at first injection, for patients after RP ± eLND (observed case) is presented in Table 2 and for patients after RT (observed case) in Table 3, respectively.

**Table 2:** Per-patient detection rate according to the PSA concentration level at first injection for patients after radical prostatectomy ± eLND (observed case) – full analysis set (N = 205)

	Per-patient detection rate [95% CI]	
PSA concentration level at first injection	[^18^F]DCFPyL N = 201	[^18^F]Choline N = 201
< 0.2 (N = 6)	2 (33.3%) [0.0;71.1]	1 (16.7%) [0.0;46.5]
[0.2–0.5] (N = 68)	24 (35.3%) [23.9;46.7]	21 (30.9%) [19.9;41.9]
[0.51–1] (N = 31)	17 (54.8%) [37.3;72.4]	10 (32.3%) [15.8;48.7]
[1.01–2] (N = 18)	13 (72.2%) [51.5;92.9]	6 (33.3%) [11.6;55.1]
> 2 (N = 9)	7 (77.8%) [50.6;100.0]	4 (44.4%) [12.0;76.9]

Observed case: missing or indeterminate results are not imputed Explanatory note: Full analysis set comprised 205 patients that had been imaged with only one tracer. Observed case refers to 201 patients that were scanned with both tracers. One-hundred-fifty out of 201 patients had radical prostatectomy ± eLND. From these, 15 patients had no PSA at baseline and 3 patients had only one tracer, resulting in a total number of 132 patients

**Table 3:** Per-patient detection rate according to the PSA concentration level at first injection for patients after radiation therapy (observed case) – full analysis set (N = 205)

	Per-patient detection rate [95% CI]	
PSA concentration level at first injection	[^18^F]DCFPyL N = 201	[^18^F]Choline N = 201
[0.2—1] (N = 0)	0 (0.0%) [0.0;0.0]	0 (0.0%) [0.0;0.0]
[1.01—2] (N = 1)	0 (0.0%) [0.0;0.0]	0 (0.0%) [0.0;0.0]
> 2 (N = 48)	43 (89.6%) [80.9;98.2]	35 (72.9%) [60.3;85.5]

Observed case: missing or indeterminate results are not imputed Explanatory note: Full analysis set comprised 205 patients that had been imaged with only 1 tracer. Observed case refers to 201 patients that were scanned with both tracers. Fifty-five out of 201 patients had radiation therapy. From these, 5 patients had no PSA at baseline and 1 patient had only one tracer, resulting in a total number of 49 patients

**Fig. 2:**
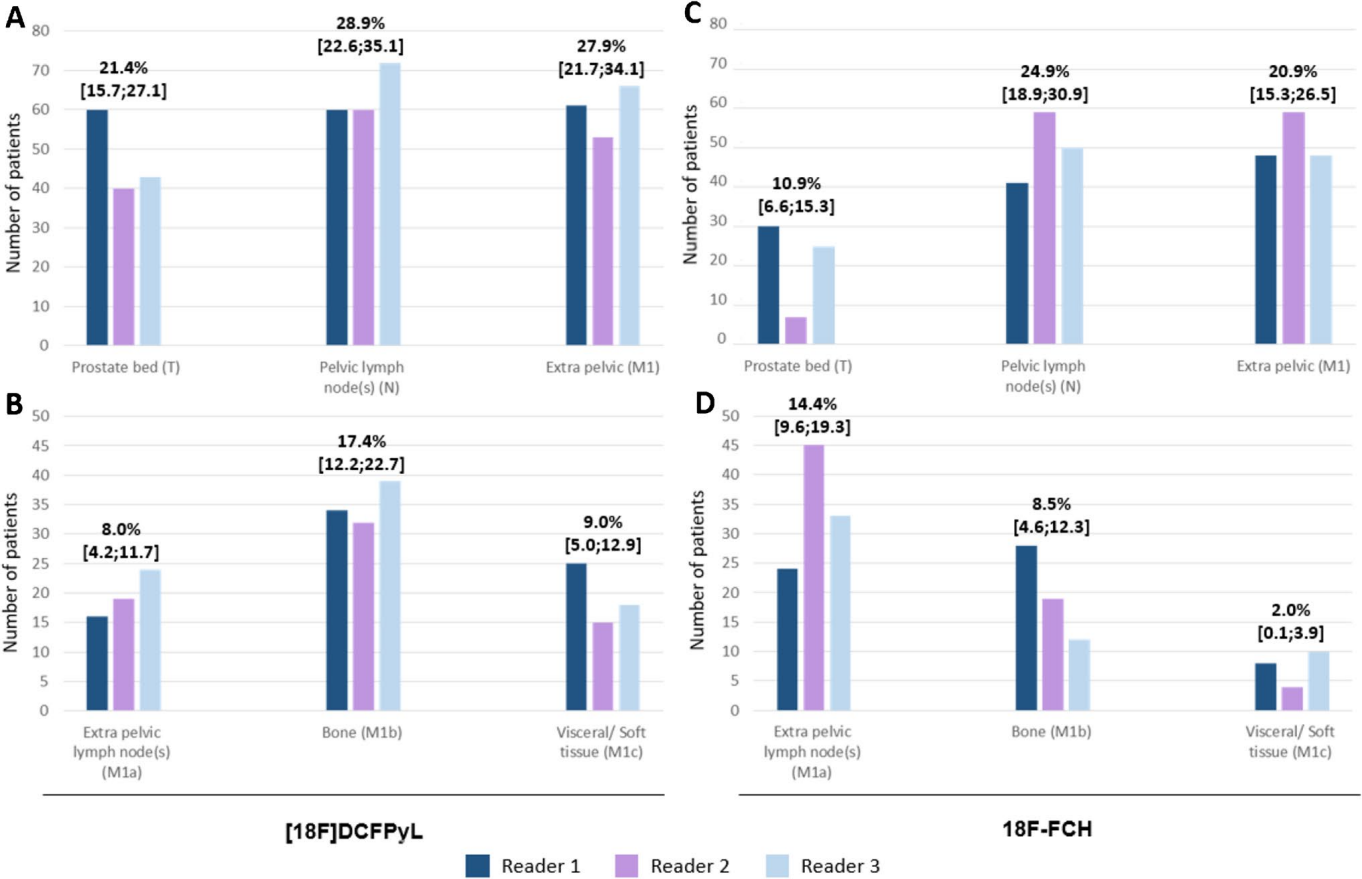
Detection rate for pelvic (**A**, **C**) and extrapelvic regions (**B**, **D**). Number of patients and detection rate (%) [95%, CI] on observed case after [18F]DCFPyL or [^18^F]fluoromethylcholine injection

In the subanalysis, local recurrences (21% vs. 11%), bone metastases (17% vs. 9%) and metastases in other organs (9.0% vs. 2.0%) were more often detected by [^18^F]DCFPyL PET/CT (all *p* < 0.001), while there was only a trend for a higher DR difference in the pelvic region (29% vs. 25%, p = 0.16) and no difference in pelvic vs. extra-pelvic lymph nodes (8.0% vs. 14%, p = 1.00). DR for pelvic and extrapelvic regions, based on observed cases, after [^18^F]DCFPyL or [^18^F]fluoromethylcholine PET/CT are presented in Fig. 2.

When considering the initial treatment with curative intent, the DR was: 47% (69/147) *versus* 30% (44/147) (*p* = 0.0002) for [^18^F]DCFPyL and [^18^F]fluoromethylcholine, respectively, in patients treated with RP ± eLND and 89% (48/54) *versus* 69% (37/54) (*p* = 0.0018) for [^18^F]DCFPyL- and [^18^F]fluoromethylcholine-PET/CT, respectively, in patients treated with RT. In addition, per-patient DR was consistent with the data reported above, when combining the level of PSA and the initial treatment with curative intent.

### Secondary endpoints

#### Impact on patient management

**Fig. 3:**
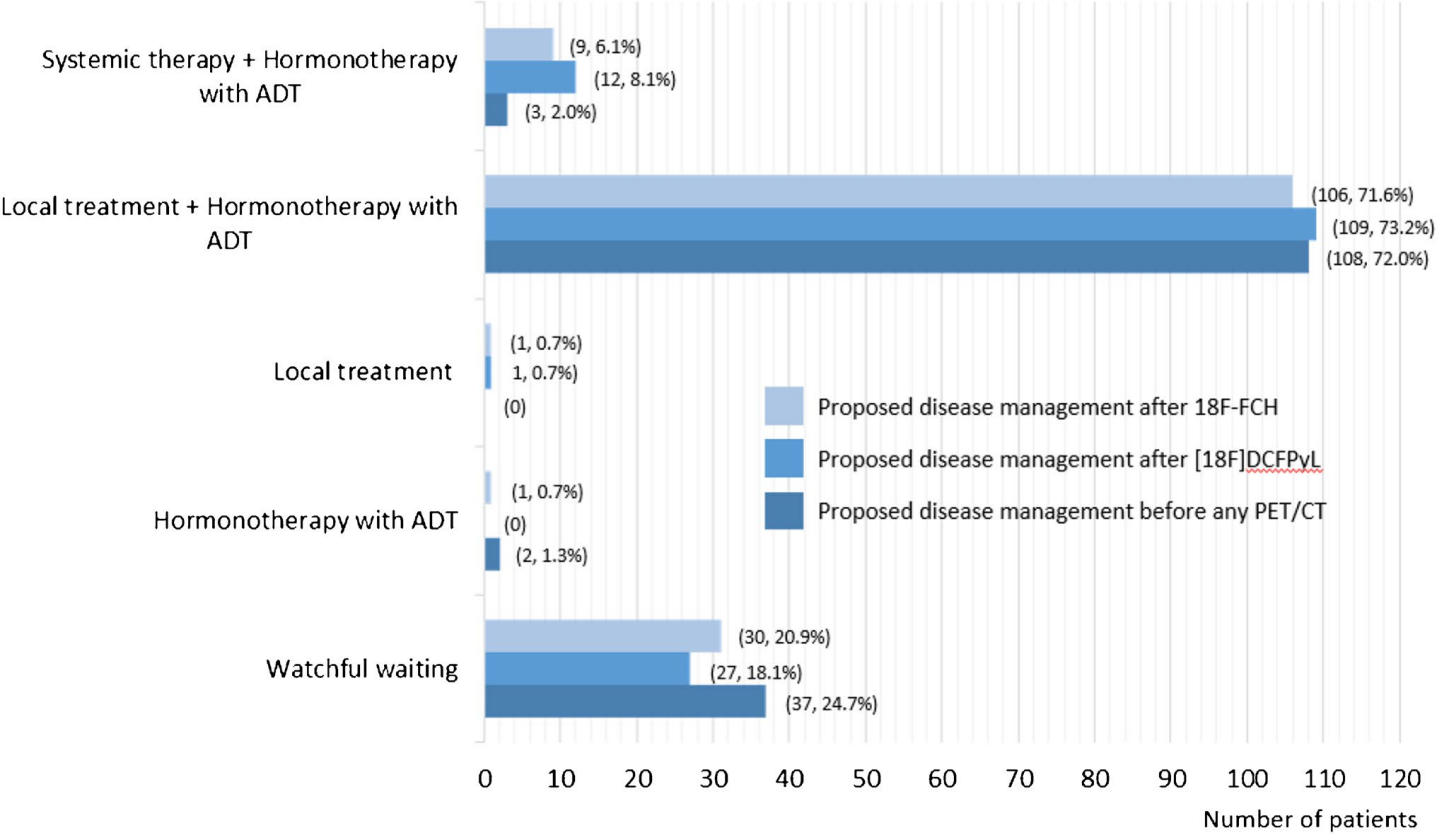
Proposed disease management by the Truth Panel in patients initially treated by radical prostatectomy with/without lymph node dissection

**Fig. 4:**
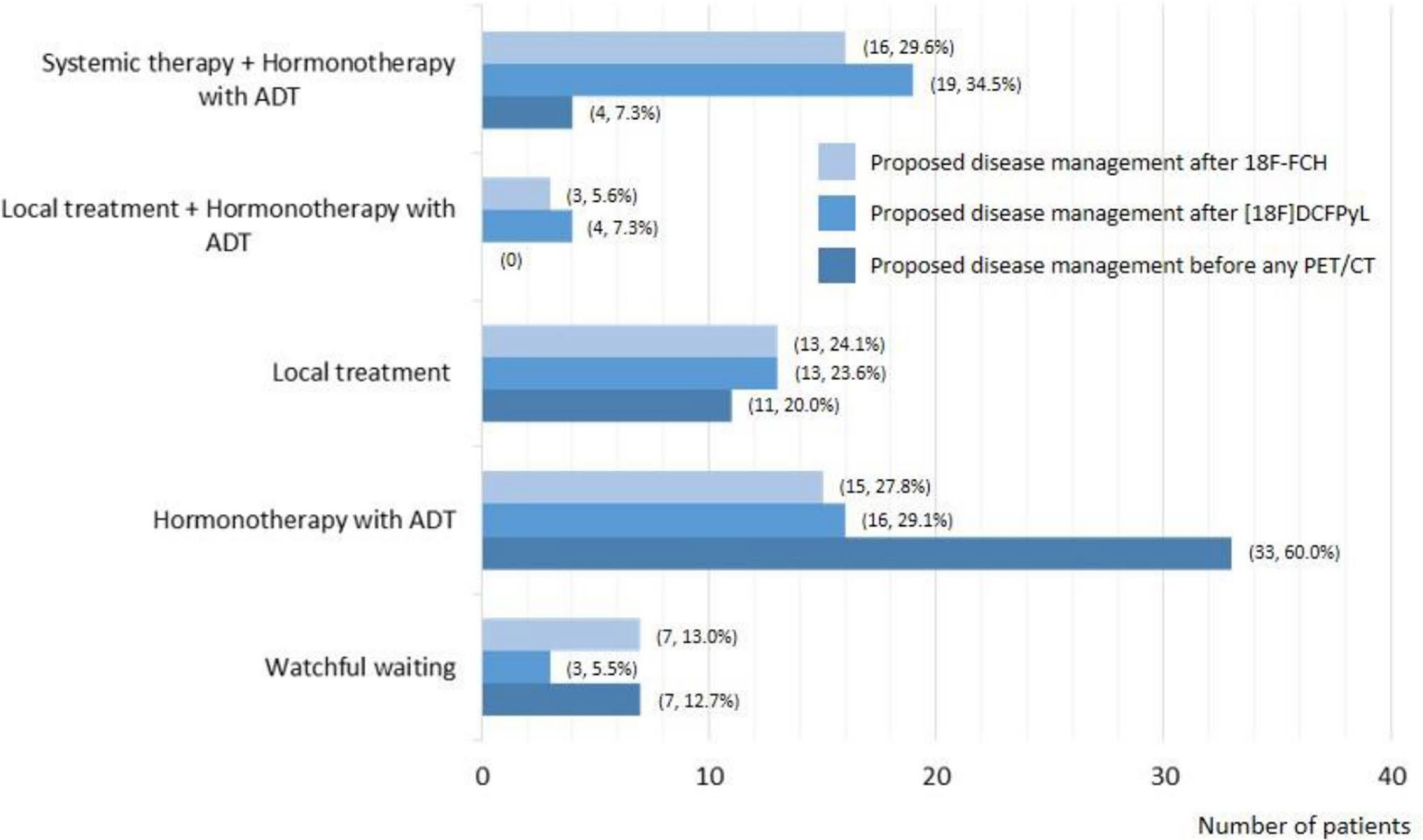
Proposed disease management by the Truth Panel in patients initially treated by curative radiation therapy

The truth panel proposed to modify treatment intent in 26.0% (N = 53 patients) and 21% (N = 43 patients) for [^18^F]DCFPyL- and [^18^F]fluoromethylcholine-PET/CT, respectively, when compared to the disease management before any PET/CT. According to the truth panel, PM proposed by the local team was impacted by the PET/CTs in 49% of the cases (100/205 patients). Among them, the impact of [^18^F]DCFPyL- and [^18^F]fluoromethylcholine-PET/CT was 44% (90/204) and 29% (58/202), respectively (Fig. 3 and Fig. 4).

**Fig. 5:**
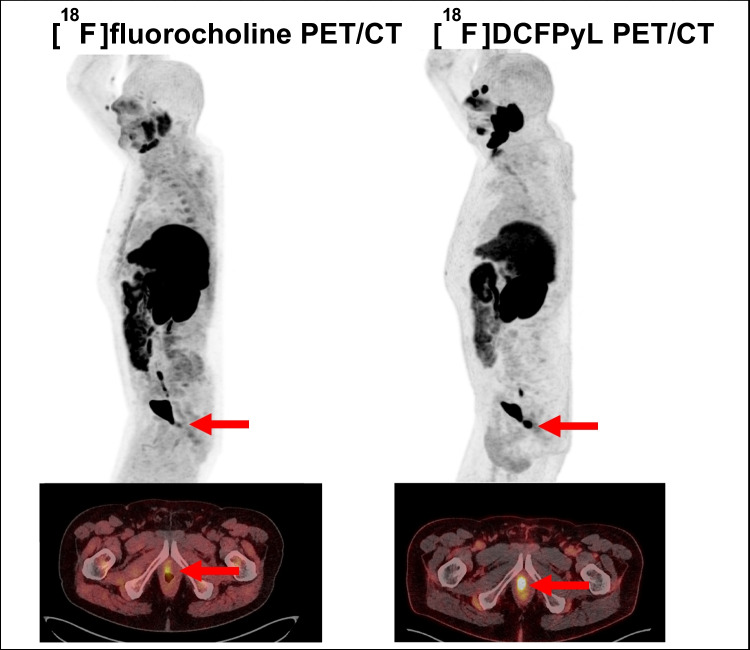
Clinical example of a patient with first prostate cancer biochemical recurrence. Sagittal Maximum Intensity Projection [^18^F]DCFPyL PET (upper right), [^18^F]fluoromethylcholine (upper left), axial fused [^18^F]DCFPyL (bottom right) and [^18^F]fluoromethylcholine PET/CT images (bottom left) of a 77-year old patient who underwent prostatectomy without lymph node dissection for ISUP grade 2 PCa, followed by an undetectable PSA level, with BCR 13 years later, at a PSA of 5.6 ng/ml. [^18^F]fluoromethylcholine -PET shows equivocal uptake in the prostate bed (SUVmax 4.1) while [^18^F]DCFPyL PET shows intense PSMA expression in the prostate bed (SUVmax 23.7). The patient underwent salvage RT of the prostate bed after PET, leading to a subsequent PSA drop to < 0.01 ng/ml

Similar results were observed when considering the initial treatment with curative intent. For RP ± eLND, a favourable management change occurred in 44.7% of cases (67/150), with an impact of the [^18^F]DCFPyL in 40.0% (60/150) versus an impact of [^18^F]fluoromethylcholine-PET/CT in 24.0% (36/150). For RT, a favourable management change occurred in 60.0% of the cases (33/55), with an impact of the [^18^F]DCFPyL in 55% (30/55) versus an impact of [^18^F]fluoromethylcholine-PET/CT in 38% (22/55). A clinical example of a patient with first prostate cancer biochemical recurrence, in whom the therapy was tailored based on the [^18^F]DCFPyL PET/CT findings, is illustrated in Fig. 5.

#### Per-region detection rate

The DR of [^18^F]DCFPyL- was significantly higher than the detection rate of [^18^F]fluoromethylcholine-PET/CT in the prostate bed, bones and other organs, but not on disease detection in the (extra)pelvic lymph nodes (Table 4). The per-region detection rate according to five pre-specified subgroups (initial treatment with curative intent, PSA level at first injection, PSA doubling time, D’Amico risk classes and ISUP grade) was barely interpretable considering the small number of patients per item evaluated. Per-region DR for the 3 different readers are presented in the Supplemental Tables 1, 2 and 3, respectively.

**Table 4:** Per-region detection rate (observed case) – full analysis set (N = 205)

	Per region detection rate [95% CI]		
Region	[^18^F]DCFPyL N = 201	[^18^F]Choline N = 201	*P*-value [a]
(T) Prostate bed	43 (21.4%) [15.7;27.1]	22 (10.9%) [6.6;15.3]	< 0.0001
(N) Pelvic lymph node(s)	58 (28.9%) [22.6;35.1]	50 (24.9%) [18.9;30.9]	0.1636
(M1) Extra pelvic	56 (27.9%) [21.7;34.1]	42 (20.9%) [15.3;26.5]	0.0285
(M1a) Extra pelvic lymph node(s)	16 (8.0%) [4.2;11.7]	29 (14.4%) [9.6;19.3]	0.9971
Retroperitoneal lymph node(s)	15 (7.5%) [3.8;11.1]	25 (12.4%) [7.9;17.0]	0.9889
Supradiaphragmatic lymph node(s)	7 (3.5%) [1.0;6.0]	6 (3.0%) [0.6;5.3]	0.5008
(M1b) Bone	35 (17.4%) [12.2;22.7]	17 (8.5%) [4.6;12.3]	0.0002
Spine	12 (6.0%) [2.7;9.3]	5 (2.5%) [0.3;4.6]	0.0194
Ribs sternum and scapula	22 (10.9%) [6.6;15.3]	10 (5.0%) [2.0;8.0]	0.0019
Iliac and sacrum	17 (8.5%) [4.6;12.3]	11 (5.5%) [2.3;8.6]	0.0553
Femur and humerus	4 (2.0%) [0.1;3.9]	3 (1.5%) [0.0;3.2]	0.5012
Others bone involvement	1 (0.5%) [0.0;1.5]	1 (0.5%) [0.0;1.5]	0.7488
(M1c) Other organ(s)	18 (9.0%) [5.0;12.9]	4 (2.0%) [0.1;3.9]	0.0003

Observed case: missing or indeterminate results are not imputed

[a] *P*-value issued from Prescott’s test

#### Safety results

Twelve Treatment Emergent Serious Adverse Events (TEAE) (6 for IMP and 6 for comparator) were reported during the study in 8/205 (3.9%) patients. Among these TEAE with the [^18^F]DCFPyL, 3/6 (50%) events were reported in the same patient. None of these TEAE were related to the [^18^F]DCFPyL or [^18^F]fluoromethylcholine and were all consistent with patient profile regarding their age, PCa stage, and medical history, notably regarding arterial hypertension. No safety signals in relation to tracer injection were reported for vital signs.

#### Inter- and intra-observer variability

Per patient DR by reader (observed case) was higher for [^18^F]DCFPyL- versus [^18^F]fluoromethylcholine- PET/CT, for all 3 readers. DR for reader 1 was 64% (129 patients) for [^18^F]DCFPyL- versus 43% (86 patients) for [^18^F]fluoromethylcholine PET/CT, while for reader 2 DR was 55% (111 patients) for [^18^F]DCFPyL- versus 41% (83 patients) for [^18^F]fluoromethylcholine -PET/CT. DR for reader 3 was 134 67% (134 patients) for [^18^F]DCFPyL- versus 44.% (89 patients) for [^18^F]fluoromethylcholine -PET/CT, respectively. The overall Cohen’s Kappa was 0.54 [95%CI 0.39; 0.68].

## Discussion

In this prospective, cross-over, open-label study, per-patient DR, impact on PM and safety profiles were evaluated and compared for [^18^F]DCFPyL- and [^18^F]fluoromethylcholine-PET/CT, in patients with first BCR PCa, after primary therapy with curative intent. Efficacy results showed greater DR and impact on PM for [^18^F]DCFPyL- compared to [^18^F]fluoromethylcholine-PET/CT. Furthermore, [^18^F]DCFPyL demonstrated a favourable toxicity profile, without any related serious AE.

PYTHON is the first comparative study, with randomized treatment administration of ‘next generation imaging’ without censorship by conventional imaging. Another phase III prospective, single-arm study, CONDOR, has evaluated the diagnostic performance and safety of [^18^F]DCFPyL in BCR PCa patients with negative/ equivocal findings on conventional imaging. [^18^F]DCFPyL was able to localize the site of BCR in 60.4%, and provided substantial PM change [20].

The diagnostic performance of [^18^F]DCFPyL was also investigated in cohort B of the prospective OSPREY trial, by comparing imaging results to histopathology. In 93 evaluable patients, median sensitivity and positive predictive value for extraprostatic lesions were 96% (95% CI: 87.8%-99.0%) and 82% (95% CI: 73.7%-90.2%), respectively. Remarkably, more than half of the patients (57.6%; 19/33) presenting with negative findings on conventional imaging, had likely distant metastases on [^18^F]DCFPyL [21].

Per-patient DR in the PYTHON study was significantly higher for [^18^F]DCFPyL than [^18^F]fluoromethylcholine-PET/CT, 58% versus 40%, respectively. This is in line with the CONDOR study, reporting [^18^F]DCFPyL PET/CT detected ≥ 1 lesion in 59% to 66% patients, as assessed by central reading. The DR between the 2 studies was comparable at low PSA values, 36% and 35% for CONDOR and PYTHON, respectively. The number of patients with PSA level < 0.5 ng/mL, was comparable in CONDOR and PYTHON (69 vs. 77 patients, respectively). Results are also consistent with the findings of Ma et al. reporting higher DR for PSMA-radiotracers, when systematically assessing BCR PCa by means of PSMA, choline, and fluciclovine-radiotracers [22].

In PYTHON, per-patient DR according to PSA concentration level at first injection for patients after RP ± eLND (observed case) tended to be higher for [^18^F]DCFPyL, across all PSA ranges (see also Supplemental Fig. 1). P values are not provided since statistics was not designed for multiplicity tests. However, no difference in DR was observed between [^18^F]DCFPyL and [^18^F]fluoromethylcholine-PET/CT for the PSA range 0.2–0.5 ng/mL. Since BCR after RP, at low PSA value, usually indicates a local relapse in prostate-bed, one explanation might be that both tracers are renally excreted. In absence of diuretics use and an early dynamic phase for [^18^F]DCFPyL, the visualization of the prostate bed might be hampered by the radioactivity in the urinary bladder.

Another explanation may be the intra-reader variability (i.e., comparison of readings made twice by a same reader), which was evaluated by the Cohen’s Kappa coefficient. The overall Cohen’s Kappa was 0.54 [95% CI 0.39; 0.68]. This low value, considered as moderate according to Landis and Koch classification, was due to reader 3. Indeed, this reader reported only a Cohen’s Kappa [95% CI] of 0.33 [0.05; 0.62]. On the contrary, reader 1 and 2 obtained a Cohen’s kappa of 0.64 [0.40; 0.88] and 0.62 [0.39; 0.84], considered as substantial. The interobserver variability of three clinically frequently used PSMA tracers in PCa was previously discussed by Hagens et al., concluding that training on interpretation is essential [23].

In a meta-analysis including 5 studies and 257 BCR PCa patients, Treglia et al. performed a head-to-head comparison between choline and PSMA in BCR PCa [24]. Overall a DR of 56% [95%CI,37–75%] for radiolabelled choline PET/CT and 78% (95% CI: 70–84%) for radiolabelled PSMA PET/CT were observed, but without a statistical significant difference at the pooled analysis and without blinded reads. Significant difference of DR was found only in patients with PSA ≥ 1 ng/ml. Radiolabelled PSMA PET/CT proved to be clearly superior in detecting BR PCa lesions, similarly to the PYTHON study.

In the PYTHON study, PM was changed by the PET/CTs in 49% of the cases, with an impact of [^18^F]DCFPyL- and [^18^F]fluoromethylcholine -PET/CT of 44% and 29%, respectively. The PM impact in the CONDOR study was 64%. The difference may be explained by the CONDOR population including mainly patients after RP + RT or RT, with negative findings on conventional imaging, while in PYTHON the patients after RT were in the minority (27%). A PM change of 60% was also described by Song et al. in an academic-center prospective evaluation of [^18^F]DCFPyL PET/CT [25].

Regarding the acquisition parameters, [^18^F]fluoromethylcholine PET/CT images were acquired 20 min p.i., following the approved image acquisition recommendations of the summary of product characteristics. [^18^F]DCFPyL PET/CT images were acquired 120 min p.i., in line with the simplified methods for quantification of the tracer, demonstrating that [^18^F]DCFPyL uptake in prostate cancer metastases rises continuously during the first 2 h after injection [26]. This finding was also supported by the observations made by Wondergem et al., who demonstrated that the detectability of metastases was higher at an uptake interval of 120 min, compared to 60 min p.i. [27].

An important aspect when visually interpreting the PSMA PET/CT scans is the non-specific bone activity. Although in present study the number of bone lesions visualised on the [^18^F]DCFPyL PET/CT scans was higher than on the [^18^F]fluroromethylcholine PET/CT, we did not report any indeterminate bone lesions. This is partially attributed to the reduced number of patients with bone lesions and the limited number of histopathologically confirmed bone metastases. However, indeterminate bone lesions on [^18^F]DCFPyL PET/CT are common, as demonstrated by Phelps et al. [28]. Therefore, the use of different parameters, like bone lesion location, intensity of the uptake, and additional scan findings is recommended since it can aid interpretation.

In this study, the intra-reader variability was low. Intra-reader variability is usually linked to deviation from study-specific image interpretation criteria on which readers have been trained, while inter-reader variability links essentially to readers’ skill. Since PET/CT readers were trained nuclear medicine physicians with track record in prostate cancer imaging, including PSMA interpretation, other possible reasons were revised for final recommendations. Our conclusion is that in order to reduce the variability, it is recommended to standardized image acquisition, to uniform the available information using the blinded setting, to standardize reader training, and to retrain the readers in case of substantial discordance is identified.

As part of the routine care practice, enrolled patients received appropriate treatment and follow-up, in a multicentre frame. The investigating sites were requested for the period of up to 10 months after the second tracer injection, to provide any results of subsequent biopsies, imaging studies, clinical findings, PSA measurements, and disease management if performed in routine practice. However, this study was not devoid of limitations. Contrary to other studies in BCR population, no standard of truth was used. Thus, treatment decisions were pragmatic, made at the discretion of the referring physician, and based on all available clinical information. Local reports of both PET/CT scans were not standardised, but followed the clinical routine, with positive findings not always being confirmed by histopathology.

## Conclusions

The per-patient detection rate of the [^18^F]DCFPyL PET/CT over [^18^F]fluoromethylcholine PET/CT was significantly higher, independent of the initial treatment with curative intent, the PSA level, the PSA doubling time, the ISUP grade or the d’Amico risk classification. In addition, [^18^F]DCFPyL PET/CT showed significantly higher per-patient sensitivity in comparison to [^18^F]fluoromethylcholine PET/CT. [^18^F]DCFPyL PET/CT had an impact on patient management in 44% of the patients, while being safe and well tolerated.

### Supplementary Information

Below is the link to the electronic supplementary material.

Supplementary file1 Supplementary Fig. 1 Clinical example of a patient with first prostate cancer biochemical recurrence. Sagittal Maximum Intensity projection [^18^F]DCFPyL PET (upper right), [^18^F]fluoromethylcholine (upper left), axial fused [^18^F]DCFPyL PET/CT images (bottom right) and [^18^F]fluoromethylcholine PET/CT images (bottom left) of a 74-year old patient who underwent prostatectomy without lymph node dissection for ISUP grade 2 PCa, followed by an undetectable PSA level, with BCR 9 years later, at a PSA of 0.25 ng/ml. [^18^F]DCFPyL PET shows intense PSMA expression (SUVmax 11.9) in a right pelvic lymph node that was negative on [^18^F]FCH PET/CT. The patient underwent salvage RT of the pelvis after PET/CT, with androgen deprivation therapy, leading to a subsequent PSA drop to < 0.01 ng/ml. (DOCX 12.9 MB)

Supplementary file2 (DOCX 15.2 KB)

Supplementary file3 (DOCX 23.6 KB)

Supplementary file4 (DOC 136 KB)

## Data Availability

The datasets generated during and/or analyzed during the current study are available from the corresponding author upon reasonable request.

## References

[1] Ferlay J, Colombet M, Soerjomataram I (2018). Cancer incidence and mortality patterns in Europe: Estimates for 40 countries and 25 major cancers in 2018. Eur J Cancer.

[2] Roehl KA, Han M, Ramos CG (2004). Cancer progression and survival rates following anatomical radical retropubic prostatectomy in 3,478 consecutive patients: long term results. J Urol.

[3] D’Amico AV, Whittington R, Malkowicz SB (2002). Biochemical outcome after radical prostatectomy or external beam radiation therapy for patients with clinically localized prostate carcinoma in the prostate specific antigen era. Cancer.

[4] Torre LA, Bray F, Siegel RL (2015). Global cancer statistics, 2012. CA Cancer J Clin.

[5] Amling CL, Blute ML, Bergstralh EJ (2000). Long-term hazard of progression after radical prostatectomy for clinically localized prostate cancer: continued risk of biochemical failure after 5 years. J Urol.

[6] Cookson MS, Aus G, Burnett AL (2007). Variation in the definition of biochemical recurrence in patients treated with localized prostate cancer: the American Urological Association (AUA) Prostate Guidelines for Localized Prostate Cancer Update Panel report and recommendation for a standard in the reporting of surgical outcomes. J Urol.

[7] Roach M, Hanks G, Thames H (2006). Defining biochemical failure following radiotherapy with or without hormonal therapy in men with clinically localized prostate cancer: recommendations of the RTOG-ASTRO Phoenix Consensus Conference. Int J Radiat Oncol Biol Phys.

[8] Giannarini G, Fossati N, Gandaglia G (2018). Will image-guided metastasis-directed therapy change the treatment paradigm of oligorecurrent prostate cancer?. Eur Urol.

[9] Habl G, Straube C, Schiller K (2017). Oligometastases from prostate cancer: local treatment with stereotactic body radiotherapy (SBRT). BMC Cancer.

[10] Tosoian JJ, Gorin MA, Ross AE, Pienta KJ, Tran PT, Schaeffer EM (2017). Oligometastatic prostate cancer: definitions, clinical outcomes, and treatment considerations. Nat Rev Urol.

[11] Cimitan M, Bortolus R, Morassut S (2006). [18F]fluoro-choline PET/CT imaging for the detection of recurrent prostate cancer at PSA relapse: experience in 100 consecutive patients. EJNMMI..

[12] Ghosh A, Heston WD (2004). Tumor target prostate specific membrane antigen (PSMA) and its regulation in prostate cancer. J Cell Biochem.

[13] Lenzo NP, Meyrick D, Turner JH (2018). Review of gallium-68 PSMA PET/CT imaging in the management of prostate cancer. Diagnostics (Basel).

[14] Rowe SP, Macura KJ, Mena E (2016). PSMA-Based [^18^F]DCFPyL PET/CT is superior to conventional imaging for lesion detection in patients with metastatic prostate cancer. Mol Imaging Biol.

[15] Zacho HD, Nielsen JB, Haberkorn U, Stenholt L, Petersen LJ (2018). 68Ga-PSMA PET/CT for the detection of bone metastases in prostate cancer: a systematic review of the published literature. Clin Physiol Funct Imaging.

[16] Mottet N, van den Bergh RC, Briers E, Van den Broeck T, Cumberbatch MG, De Santis M, Fanti S, Fossati N, Gandaglia G, Gillessen S, Grivas N. EAU-EANM-ESTRO-ESUR-SIOG guidelines on prostate cancer—2020 update. Part 1: screening, diagnosis, and local treatment with curative intent. European Urology. 2021;79(2):243–62. 10.1016/j.eururo.2020.09.042. 10.1016/j.eururo.2020.09.042 33172724

[17] Giesel FL, Will L, Lawal I (2018). Intraindividual comparison of ^18^F-PSMA-1007 and ^18^F-DCFPyL PET/CT in the prospective evaluation of patients with newly diagnosed prostate carcinoma: a pilot study. J Nucl Med.

[18] Gorin MA, Rowe SP, Patel HD (2018). Prostate specific membrane antigen targeted ^18^FDCFPyL positron emission tomography/computerized tomography for the preoperative staging of high risk prostate cancer: results of a prospective, phase II, single center study. J Urol.

[19] Szabo Z, Mena E, Rowe SP (2015). Initial evaluation of [^18^F]DCFPyL for prostate-specific membrane antigen (PSMA)-targeted PET imaging of prostate cancer. Mol Imaging Biol.

[20] Morris M, Rowe SP, Gorin MA (2021). Diagnostic Performance of 18F-DCFPyL-PET/CT in Men with Biochemically Recurrent Prostate Cancer: Results from the CONDOR Phase III. Multicenter Study Clin Cancer Res.

[21] Pienta KJ, MA, Rowe SP,  (2021). A Phase 2/3 Prospective Multicenter Study of the Diagnostic Accuracy of Prostate Specific Membrane Antigen PET/CT with 18F-DCFPyL in Prostate Cancer Patients (OSPREY). Urol..

[22] Ma W, Mao J, Yang J (2022). Comparing the diagnostic performance of radiotracers in prostate cancer biochemical recurrence: a systematic review and meta-analysis. Eur Radiol.

[23] Hagens MJ, Oprea-Lager DE, Vis AN (2022). Reproducibility of PSMA PET/CT Imaging for Primary Staging of Treatment-Naïve Prostate Cancer Patients Depends on the Applied Radiotracer: A Retrospective Study. J Nucl Med.

[24] Treglia G, Pereira Mestre R, Ferrari M (2019). Radiolabelled choline versus PSMA PET/CT in prostate cancer restaging: a meta-analysis. Am J Nucl Med Mol Imaging.

[25] Song H, Harrison C, Duan H (2020). Prospective Evaluation of 18F-DCFPyL PET/CT in Biochemically Recurrent Prostate Cancer in an Academic Center: A Focus on Disease Localization and Changes in Management. J Nucl Med.

[26] Jansen BHE, Yaqub M, Voortman J (2019). Simplified Methods for Quantification of 18F-DCFPyL Uptake in Patients with Prostate Cancer. J Nucl Med..

[27] Wondergem M, van der Zant FM, Knol RJJ (2017). 18F-DCFPyL PET/CT in the Detection of Prostate Cancer at 60 and 120 Minutes: Detection Rate, Image Quality, Activity Kinetics, and Biodistribution. J Nucl Med..

[28] Phelps TE, Harmon SA, Mena E (2023). Predicting Outcomes of Indeterminate Bone Lesions on 18F-DCFPyL PSMA PET/CT Scans in the Setting of High-Risk Primary or Recurrent Prostate Cancer. J Nucl Med..

