# Supplementary material for: [18F]DCFPyL PET/CT versus [18F]fluoromethylcholine PET/CT in Biochemical Recurrence of Prostate Cancer (PYTHON): a prospective, open label, cross-over, comparative study
Source: Eur J Nucl Med Mol Imaging. 2023 Jun 21;50(11):3439–51. doi: 10.1007/s00259-023-06301-5 (PMC10542307; doi:10.1007/s00259-023-06301-5)
Supplement: Supplementary file 1 — Supplementary file1 Supplementary Fig. 1 Clinical example of a patient with first prostate cancer biochemical recurrence. Sagittal Maximum Intensity projection [18F]DCFPyL PET (upper right), [18F]fluoromethylcholine (upper left), axial fused [18F]DCFPyL PET/CT images (bottom right) and [18F]fluoromethylcholine PET/CT images (bottom left) of a 74-year old patient who underwent prostatectomy without lymph node dissection for ISUP grade 2 PCa, followed by an undetectable PSA level, with BCR 9 years later, at a PSA of 0.25 ng/ml. [18F]DCFPyL PET shows intense PSMA expression (SUVmax 11.9) in a right pelvic lymph node that was negative on [18F]FCH PET/CT. The patient underwent salvage RT of the pelvis after PET/CT, with androgen deprivation therapy, leading to a subsequent PSA drop to < 0.01 ng/ml. (DOCX 12.9 MB) [file 259_2023_6301_MOESM1_ESM.docx]

**Supplemental Fig. 1** Clinical example of a patient with first prostate cancer biochemical recurrence. (A) Sagittal Maximum Intensity projection [^18^F]DCFPyL PET (upper right), (B) [^18^F]fluoromethylcholine (upper left) and axial fused [^18^F]DCFPyL PET/CT images (bottom right) and [18F]fluoromethylcholine PET/CT images (bottom left) of a 74-year old patient who underwent prostatectomy without lymph node dissection for ISUP grade 2 PCa, followed by an undetectable PSA level, with BCR 9 years later, at a PSA of 0.25 ng/ml. [^18^F]DCFPyL PET shows intense PSMA expression (SUVmax 11.9) in a right pelvic lymph node that was negative on [^18^F]FCH PET/CT. The patient underwent salvage RT of the pelvis after PET/CT, with androgen deprivation therapy, leading to a subsequent PSA drop to <0.01 ng/ml.


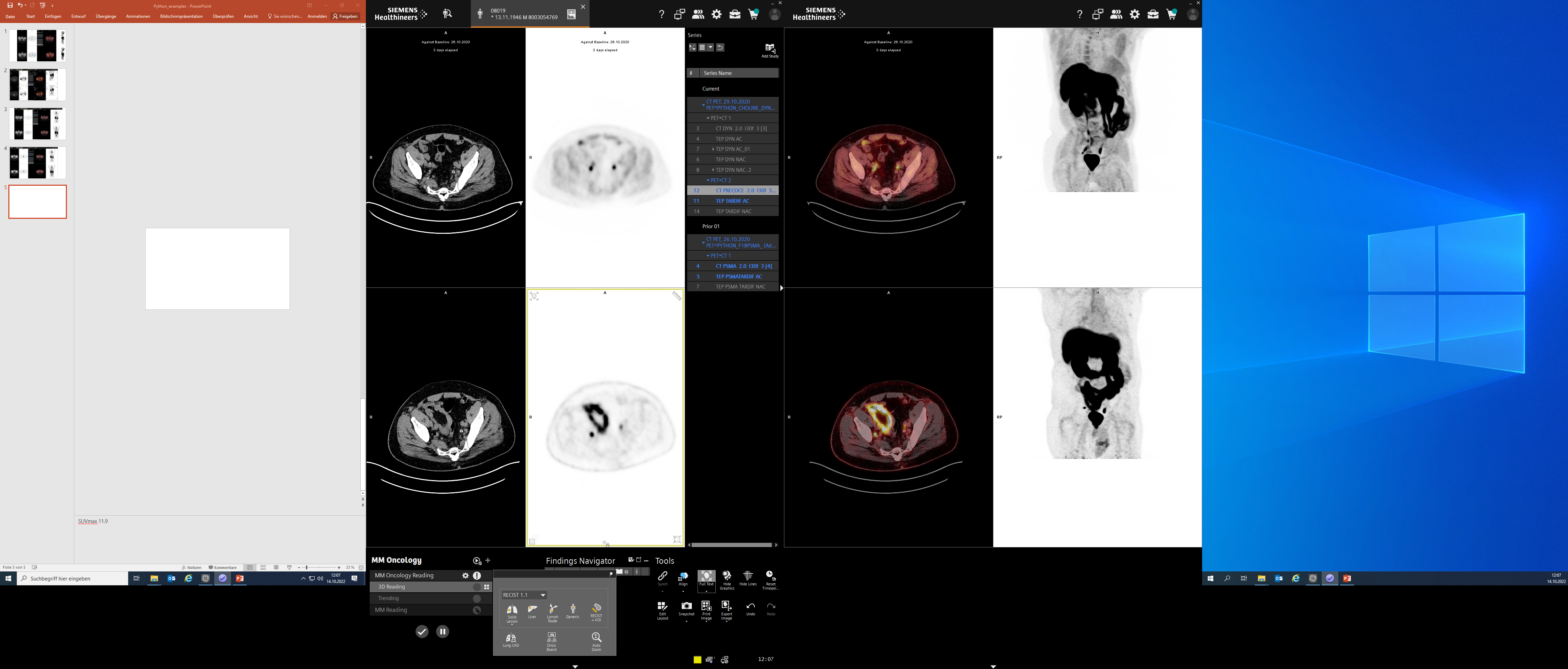

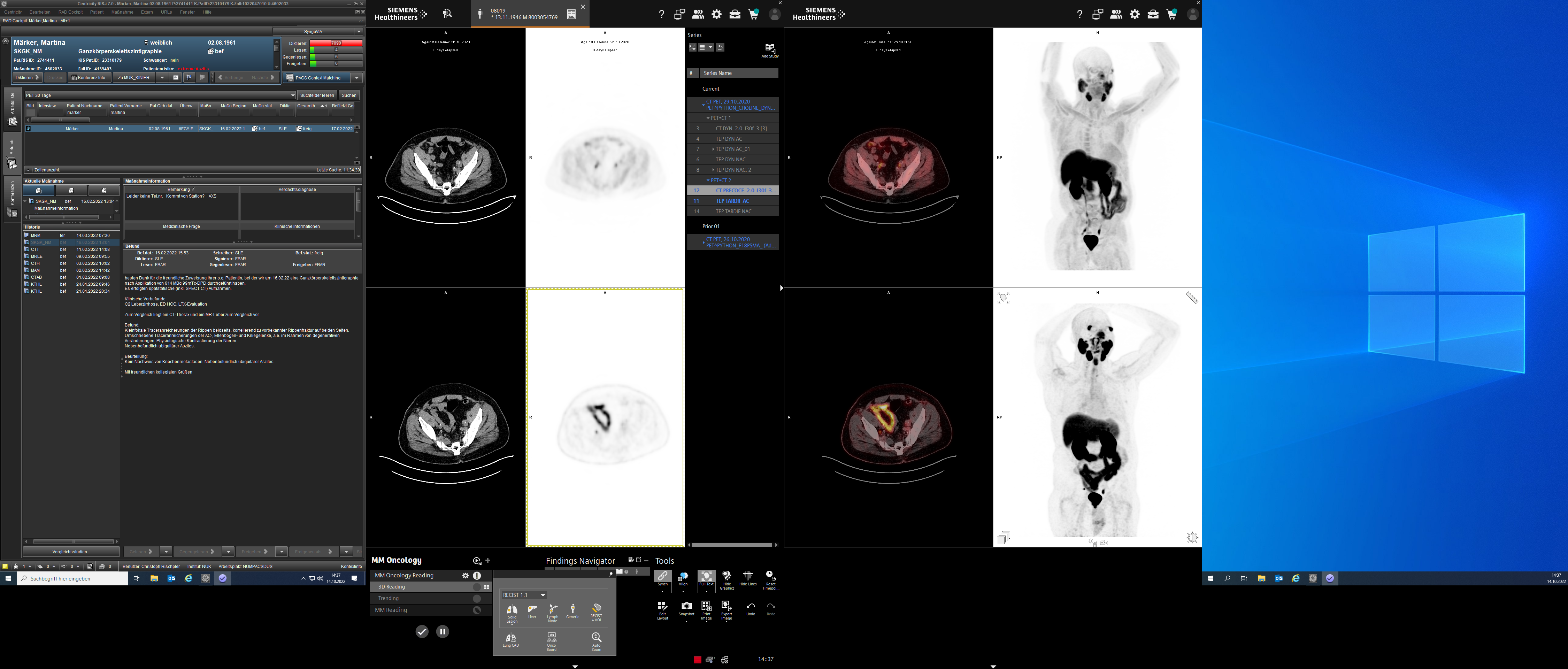


**[^18^F]fluorocholine PET/CT**


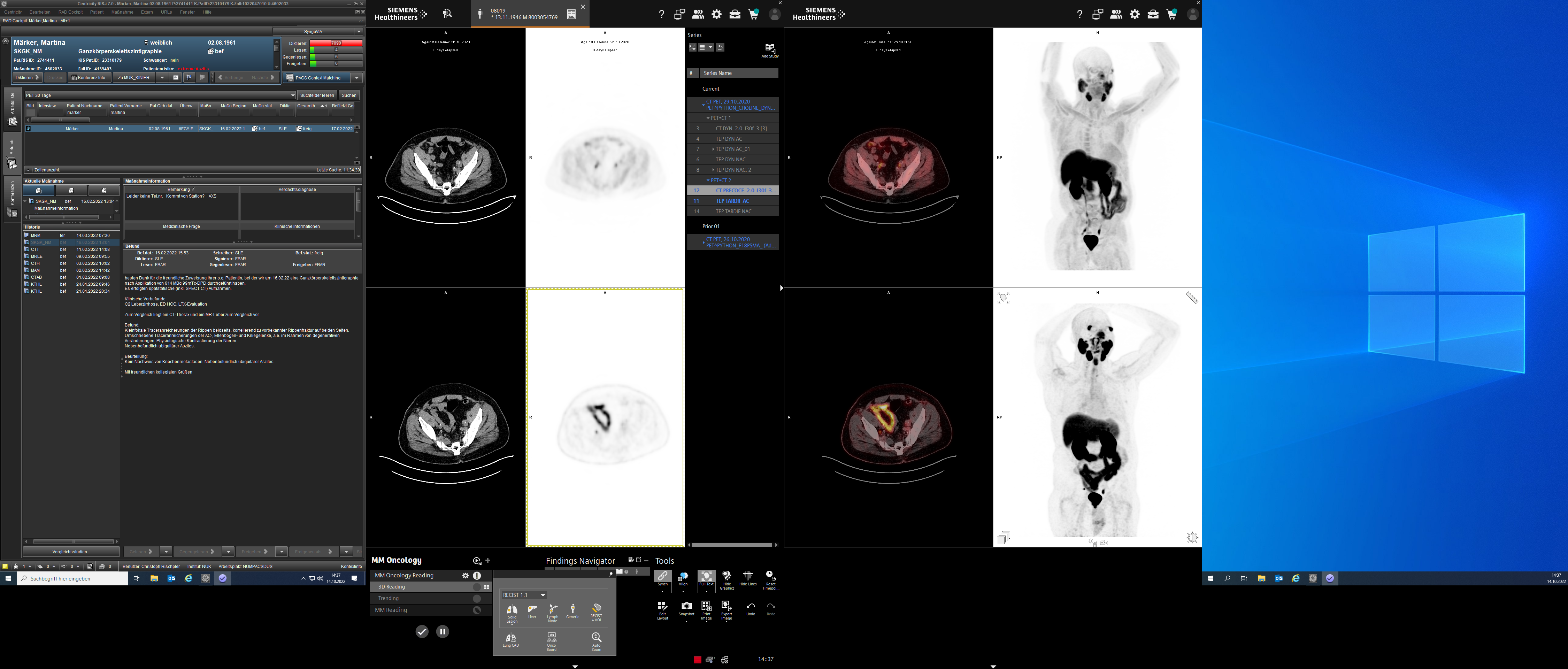

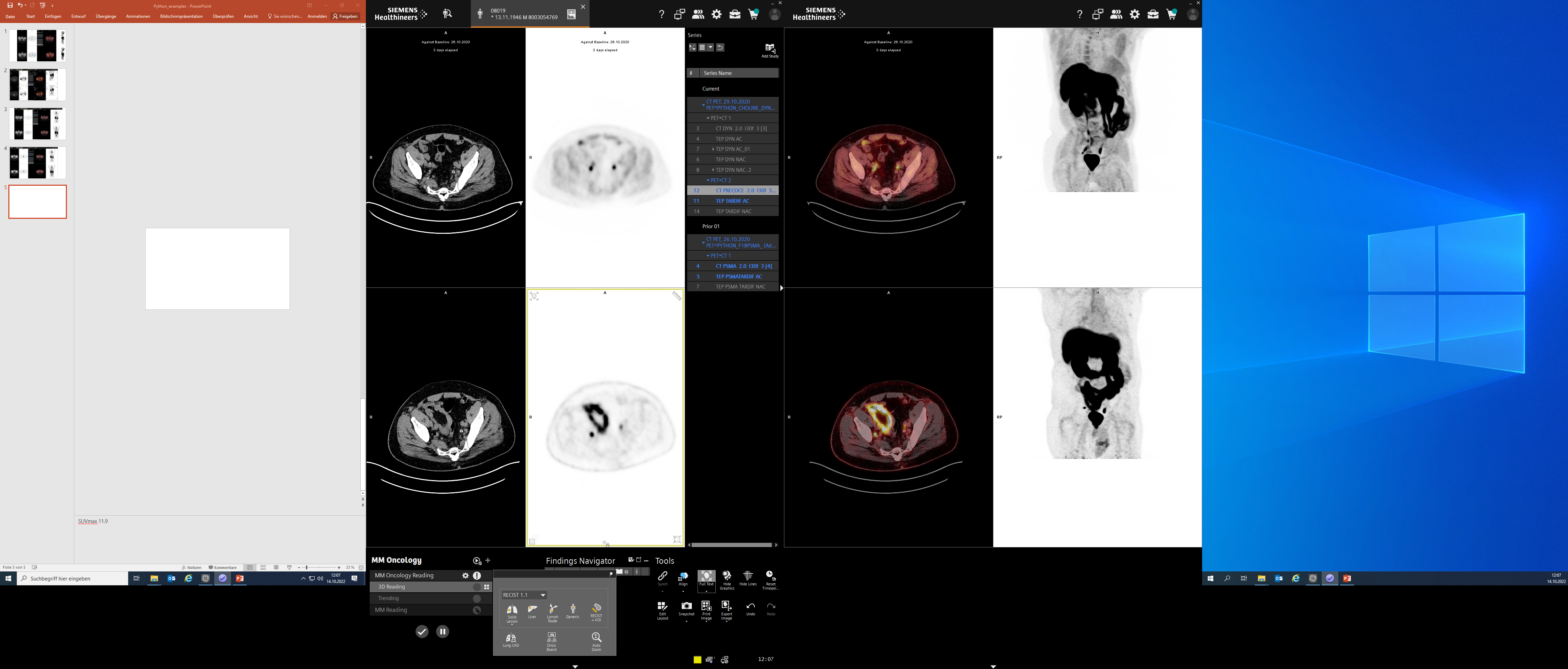


**[^18^F]DCFPyL PET/CT**

**A**

**B**
