# Supplementary material for: [18F]DCFPyL PET/CT versus [18F]fluoromethylcholine PET/CT in Biochemical Recurrence of Prostate Cancer (PYTHON): a prospective, open label, cross-over, comparative study
Source: Eur J Nucl Med Mol Imaging. 2023 Jun 21;50(11):3439–51. doi: 10.1007/s00259-023-06301-5 (PMC10542307; doi:10.1007/s00259-023-06301-5)
Supplement: Supplementary file 2 — Supplementary file2 (DOCX 15.2 KB) [file 259_2023_6301_MOESM2_ESM.docx]

**Supplementary file 2**

**Safety analysis**

Patients were asked for any adverse events (AEs) occurring from the injection up to 24 hours following each radiopharmaceutical injection. These AEs were recorded in the electronic clinical research form (e-CRF). Any serious adverse event occurring after the 24h-follow-up period and stated as related to any of the ^18^F-tracers by the reporting physician, were recorded in the e-CRF, and sent on the Serious Adverse Event (SAE) Report Form to CURIUM Drug Safety unit. Only Treatment-Emergent Adverse Events (TEAEs), defined as any AE with an onset date on or within the 24 hours following the first injection of the study treatment ([^18^F]DCFPyL / ^18^F-FCH) or any ongoing event that worsens within the 24 hours following the first injection of the study treatment, were analysed. Other AE were listed. The incidence of overall TEAEs, Drug-Related TEAEs, SAEs, and Treatment Emergent Serios Adverse Events (TESAEs) leading to treatment discontinuation were provided for each study treatment. The Medical Dictionary for Regulatory Activities (MedDRA) was used for the classification of AEs. TEAEs were summarised by MedDRA System Organ Class (SOC) and Preferred Terms (PT) for each study product. Other safety endpoints such as vital signs were descriptively summarised.

The AE term (verbatim term) was assigned to the lowest level term (LLT), and a preferred term (PT) was classified by a high-level term (HLT), a high-level group term (HLGT) and a system organ class (SOC) according to the MedDRA thesaurus version 24.1.

TEAEs were defined as any AE starting at the time or within the 24 hours following the start of injection (of [^18F^]DCFPyL or ^18^F-FCH).

The number of AEs and the percentage of patients who had at least one AE were described as follows, separately for [^18F^]DCFPyL and ^18^F-FCH:

•Treatment-emergent adverse events (TEAEs);

•Treatment-emergent serious adverse events (TESAEs);

•Drug-related TEAEs;

•TEAEs leading to treatment discontinuation.

An event was considered as related to the study treatment if the relationship is ticked “Possible”, “Probable” or “Definite”.

All these categories of events were presented by SOC and PT separately for [^18^F]DCFPyL and ^18^F-FCH, seconded by a list including the following variables: patient identifier, age at baseline, tracer, dates of tracer injection, nature of the AE (verbatim), SOC, PT, onset date/time, end date/time/ongoing, seriousness, severity, relatedness to the study drugs, action taken (if applicable), outcome of event, was also recorded when applicable.
