# Supplementary material for: [18F]DCFPyL PET/CT versus [18F]fluoromethylcholine PET/CT in Biochemical Recurrence of Prostate Cancer (PYTHON): a prospective, open label, cross-over, comparative study
Source: Eur J Nucl Med Mol Imaging. 2023 Jun 21;50(11):3439–51. doi: 10.1007/s00259-023-06301-5 (PMC10542307; doi:10.1007/s00259-023-06301-5)
Supplement: Supplementary file 3 — Supplementary file3 (DOCX 23.6 KB) [file 259_2023_6301_MOESM3_ESM.docx]

**Supplementary file 3**

**Statistical analyses**

Analyses were performed mainly as descriptive statistics and were summarised depending on the nature of the analysed variables. In addition, 95% confidence intervals (CI) were calculated when appropriate. Unless otherwise specified, these intervals were two-sided and provide 95% confidence. By default, the Agresti-Coull 95%CI was provided for discrete variables and the Wald 95%CI for continuous variables.

*Primary efficacy endpoints analysis*

The analysis of the primary objective was performed in the FAS. Any missing data regarding the detection status for one of the two study PET/CTs (injection not performed, missing images) or PET/CT results that were indeterminate were imputed to “Negative” (*i.e.,* defined as the “worst case” imputation). The detection status without imputation was also presented, including the reason of missing data. The Prescott’s test was used to assess the difference between the two methods in term of detection. Moreover, several analyses were performed:

- on the FAS, any missing data regarding the detection status for one of the two PET/CTs was imputed to “Positive” (*i.e*., defined as the “best case” imputation)
- on the FAS, only “observed cases” were taken into account (*i.e*., no imputation was performed, and was considered as “assessed” in all patients with at least an evaluation by one of the independent readers)
- on the PP set, only “observed cases” were planned to be considered. This analysis was planned to be carried out if the number of patients of the PP set differed by more than 10% from the FAS “observed case” set which was not the case.

The per-patient detection rate was computed for each reader independently and the intra-reader variability and inter-reader variability was evaluated by the Cohen’s kappa coefficient and the Fleiss’ kappa coefficient, respectively, on the FAS (without any imputation). No multiplicity adjustment was performed for the defined analyses.

*Secondary endpoints analyses*

Per-region detection rates were analysed on the FAS in the same way as the per-patient detection rates.

The sensitivity and the specificity of [^18^F]DCFPyL PET/CT and of ^18^F-FCH PET/CT to detect recurrence of PCa were determined with the associated two-sided 95%CI on a per-patient basis and on a per-region basis in reference to the composite SOR. The sensitivities and specificities were compared between [^18^F]DCFPyL PET/CT and ^18^F-FCH PET/CT using a McNemar chi2 test “revisited”. Per-patient and per-region sensitivity and specificity were also computed for each reader separately.

The concordance rate between [^18^F]DCFPyL PET/CT and ^18^F-FCH PET/CT per-region were computed as well as the corresponding two-sided 95%CI. The per-region concordance rate was defined as the ratio between (the number of regions defined as positive by both [^18^F]DCFPyL PET/CT and ^18^F-FCH PET/CT) + (the number of regions defined as negative by both [^18^F]DCFPyL PET/CT and ^18^F-FCH PET/CT) and the total number of assessed regions.

The clinical impact of [^18^F]DCFPyL PET/CT and ^18^F-FCH PET/CT on disease status and proposed management was evaluated by the truth panel. First, the truth panel determined management based on baseline data without knowing the results of the PET/CTs. Impact on patient management was then assessed by the truth panel in two different ways: i) by proposing a patient management after each of the individual PET/CTs (PET/CT being proposed blindly) and ii) by assessing the treatment implemented locally by the investigator who considered the common information of the two PET/CTs at the time of the choice of therapeutic management. The percentage of changes between the proposed management at baseline and the proposed management after each PET/CT represents the impact of each of the PETs on patient management.
