# Supplementary material for: [18F]DCFPyL PET/CT versus [18F]fluoromethylcholine PET/CT in Biochemical Recurrence of Prostate Cancer (PYTHON): a prospective, open label, cross-over, comparative study
Source: Eur J Nucl Med Mol Imaging. 2023 Jun 21;50(11):3439–51. doi: 10.1007/s00259-023-06301-5 (PMC10542307; doi:10.1007/s00259-023-06301-5)
Supplement: Supplementary file 4 — Supplementary file4 (DOC 136 KB) [file 259_2023_6301_MOESM4_ESM.doc]

## **Supplementary Table 1** Per-region detection rate for reader 1 (observed case) – FAS (N=205)

**Page 1 of 3**

|  | **Per region detection rate [95% CI]** | |  |
| --- | --- | --- | --- |
| **Region** | **18F-DCFPyL N = 201** | **18F-FCH N = 201** | **P-value [a]** |
|  |  |  |  |
| (T) Prostate bed | 60 (29.9%) [23.5;36.2] | 30 (14.9%) [10.0;19.9] | <0.0001 |
|  |  |  |  |
| (N) Pelvic lymph node(s) | 60 (29.9%) [23.5;36.2] | 41 (20.4%) [14.8;26.0] | 0.0048 |
|  |  |  |  |
| (M1) Extra pelvic | 61 (30.3%) [24.0;36.7] | 48 (23.9%) [18.0;29.8] | 0.0345 |
|  |  |  |  |
| (M1a) Extra pelvic lymph node(s) | 16 (8.0%) [4.2;11.7] | 24 (11.9%) [7.5;16.4] | 0.9621 |
|  |  |  |  |
| Retroperitoneal lymph node(s) | 15 (7.5%) [3.8;11.1] | 21 (10.4%) [6.2;14.7] | 0.9335 |
|  |  |  |  |
| Supradiaphragmatic lymph node(s) | 8 (4.0%) [1.3;6.7] | 7 (3.5%) [1.0;6.0] | 0.5020 |
|  |  |  |  |
| (M1b) Bone | 34 (16.9%) [11.7;22.1] | 28 (13.9%) [9.1;18.7] | 0.1653 |
|  |  |  |  |
| Spine | 12 (6.0%) [2.7;9.3] | 13 (6.5%) [3.1;9.9] | 0.7450 |
|  |  |  |  |
| Ribs sternum and scapula | 21 (10.4%) [6.2;14.7] | 11 (5.5%) [2.3;8.6] | 0.0026 |
|  |  |  |  |
| Iliac and sacrum | 19 (9.5%) [5.4;13.5] | 19 (9.5%) [5.4;13.5] | 0.5979 |
|  |  |  |  |
| Femurs and humerus | 6 (3.0%) [0.6;5.3] | 6 (3.0%) [0.6;5.3] | 0.6558 |
|  |  |  |  |
| Others bone involvement | 4 (2.0%) [0.1;3.9] | 2 (1.0%) [0.0;2.4] | 0.3442 |
|  |  |  |  |
| (M1c) Other organ(s) | 25 (12.4%) [7.9;17.0] | 8 (4.0%) [1.3;6.7] | 0.0001 |

Observed case: missing or indeterminate results are not imputed

[a] P-value issued from Prescott’s test

Database: ADIMG, Database lock date: 21FEB2022, Program: Detection_rate.sas, Generated on 11APR2023 10:23

## **Supplementary Table 1** Per-region detection rate for reader 2 (observed case) – FAS (N=205**)**

**Page 2 of 3**

|  | **Per region detection rate [95% CI]** | |  |
| --- | --- | --- | --- |
| **Region** | **18F-DCFPyL N = 201** | **18F-FCH N = 201** | **P-value [a]** |
|  |  |  |  |
| (T) Prostate bed | 40 (19.9%) [14.4;25.4] | 7 (3.5%) [1.0;6.0] | <0.0001 |
|  |  |  |  |
| (N) Pelvic lymph node(s) | 60 (29.9%) [23.5;36.2] | 59 (29.4%) [23.1;35.7] | 0.4976 |
|  |  |  |  |
| (M1) Extra pelvic | 53 (26.4%) [20.3;32.5] | 59 (29.4%) [23.1;35.7] | 0.8387 |
|  |  |  |  |
| (M1a) Extra pelvic lymph node(s) | 19 (9.5%) [5.4;13.5] | 45 (22.4%) [16.6;28.2] | 1 |
|  |  |  |  |
| Retroperitoneal lymph node(s) | 18 (9.0%) [5.0;12.9] | 45 (22.4%) [16.6;28.2] | 1 |
|  |  |  |  |
| Supradiaphragmatic lymph node(s) | 7 (3.5%) [1.0;6.0] | 2 (1.0%) [0.0;2.4] | 0.0314 |
|  |  |  |  |
| (M1b) Bone | 32 (15.9%) [10.9;21.0] | 19 (9.5%) [5.4;13.5] | 0.0054 |
|  |  |  |  |
| Spine | 14 (7.0%) [3.5;10.5] | 6 (3.0%) [0.6;5.3] | 0.0192 |
|  |  |  |  |
| Ribs sternum and scapula | 18 (9.0%) [5.0;12.9] | 10 (5.0%) [2.0;8.0] | 0.0108 |
|  |  |  |  |
| Iliac and sacrum | 16 (8.0%) [4.2;11.7] | 12 (6.0%) [2.7;9.3] | 0.1731 |
|  |  |  |  |
| Femur and humerus | 4 (2.0%) [0.1;3.9] | 4 (2.0%) [0.1;3.9] | 0.6893 |
|  |  |  |  |
| Others bone involvement | 1 (0.5%) [0.0;1.5] | 1 (0.5%) [0.0;1.5] | 0.7488 |
|  |  |  |  |
| (M1c) Other organ(s) | 15 (7.5%) [3.8;11.1] | 4 (2.0%) [0.1;3.9] | 0.0005 |

Observed case: missing or indeterminate results are not imputed

[a] P-value issued from Prescott’s test

Database: ADIMG, Database lock date: 21FEB2022, Program: Detection_rate.sas, Generated on 11APR2023 10:23

## **Supplementary Table 3** Per-region detection rate for reader 3 (observed case) – FAS (N=205)

**Page 3 of 3**

|  | **Per region detection rate [95% CI]** | |  |
| --- | --- | --- | --- |
| **Region** | **18F-DCFPyL N = 201** | **18F-FCH N = 201** | **P-value [a]** |
|  |  |  |  |
| (T) Prostate bed | 43 (21.4%) [15.7;27.1] | 25 (12.4%) [7.9;17.0] | 0.0003 |
|  |  |  |  |
| (N) Pelvic lymph node(s) | 72 (35.8%) [29.2;42.5] | 50 (24.9%) [18.9;30.9] | 0.0032 |
|  |  |  |  |
| (M1) Extra pelvic | 66 (32.8%) [26.3;39.3] | 48 (23.9%) [18.0;29.8] | 0.0096 |
|  |  |  |  |
| (M1a) Extra pelvic lymph node(s) | 24 (11.9%) [7.5;16.4] | 33 (16.4%) [11.3;21.5] | 0.9596 |
|  |  |  |  |
| Retroperitoneal lymph node(s) | 20 (10.0%) [5.8;14.1] | 26 (12.9%) [8.3;17.6] | 0.9260 |
|  |  |  |  |
| Supradiaphragmatic lymph node(s) | 11 (5.5%) [2.3;8.6] | 12 (6.0%) [2.7;9.3] | 0.6878 |
|  |  |  |  |
| (M1b) Bone | 39 (19.4%) [13.9;24.9] | 12 (6.0%) [2.7;9.3] | <0.0001 |
|  |  |  |  |
| Spine | 10 (5.0%) [2.0;8.0] | 3 (1.5%) [0.0;3.2] | 0.0077 |
|  |  |  |  |
| Ribs sternum and scapula | 25 (12.4%) [7.9;17.0] | 8 (4.0%) [1.3;6.7] | 0.0001 |
|  |  |  |  |
| Iliac and sacrum | 18 (9.0%) [5.0;12.9] | 8 (4.0%) [1.3;6.7] | 0.0032 |
|  |  |  |  |
| Femur and humerus | 2 (1.0%) [0.0;2.4] | 2 (1.0%) [0.0;2.4] | 1 |
|  |  |  |  |
| Others bone involvement | 0 (0.0%) [0.0;0.0] | 0 (0.0%) [0.0;0.0] | 1 |
|  |  |  |  |
| (M1c) Other organ(s) | 18 (9.0%) [5.0;12.9] | 10 (5.0%) [2.0;8.0] | 0.0287 |

Observed case: missing or indeterminate results are not imputed

[a] P-value issued from Prescott’s test

Database: ADIMG, Database lock date: 21FEB2022, Program: Detection_rate.sas, Generated on 11APR2023 10:23
